# Study Progression of Apelin/APJ Signaling and Apela in Different Types of Cancer

**DOI:** 10.3389/fonc.2021.658253

**Published:** 2021-04-12

**Authors:** Longfei Liu, Xiaoping Yi, Can Lu, Yong Wang, Qiao Xiao, Liang Zhang, Yingxian Pang, Xiao Guan

**Affiliations:** ^1^ Department of Urology, Xiangya Hospital, Central South University, Changsha, China; ^2^ Department of Radiology, Xiangya Hospital, Central South University, Changsha, China; ^3^ Department of Nephrology, The Second Xiangya Hospital of Central South University, Changsha, China

**Keywords:** apelin, APJ, Apela, cancer, therapeutic targets

## Abstract

Apelin is an endogenous ligand that binds to the G protein-coupled receptor angiotensin-like-receptor 1 (APJ). Apelin and APJ are widely distributed in organs and tissues and are involved in multiple physiological and pathological processes including cardiovascular regulation, neuroendocrine stress response, energy metabolism, etc. Additionally, apelin/APJ axis was found to play an important role in cancer development and progression. Apela is a newly identified endogenous ligand for APJ. Several studies have revealed the potential role of Apela in cancers. In this article, we review the current studies focusing on the role of apelin/APJ signaling and Apela in different cancers. Potential mechanisms by which apelin/APJ and Apela mediate the regulation of cancer development and progression were also mentioned. The Apelin/APJ signaling and Apela may serve as potential therapeutic candidates for treatment of cancer.

## Introduction

Apelin is a bioactive peptide isolated from bovine stomach in 1998 and is recognized as the endogenous ligand for angiotensin-like-receptor 1 (APJ), which belongs to the G protein coupled receptor family (1, 2). The apelin gene (APLN) in human is located on chromosome Xq25-q26.1. The APLN encodes a secreted precursor called preproapelin consisting of 77 amino acids. The preproapein is cleaved by endopetidases, generating several active forms of apelin including apelin-12, -13, -17, -36, and other (3–5). Among these forms, it has been discovered that apelin-13 and apelin-17 show stronger activity than apelin-36. The APJ gene is located on chromosome 11q12 and encodes a 380-amino acid protein. APJ is a G protein coupled receptor with 7 transmembrane domains which was first identified in 1993. It shows a close sequence homology to the angiotensin II receptor type 1 (6). However, APJ cannot bind Ang II. In human, the apelin/APJ axis is widely expressed in organs like heart, brain, kidney, placenta, etc. (7, 8). It has been demonstrated that the apelin/APJ system plays a pivotal role in multiple physiological processes including cardiovascular regulation, angiogenesis, pain, energy metabolism, fluid homeostasis, gastrointestinal function, neuroendocrine stress response and feeding behavior (4, 9–15).

Recently, a new endogenous peptide that binds to APJ was discovered. Apelin early ligand A (APELA), also known as Elabela, Toddler, and ELA, is an evolutionarily conserved peptide hormone that was firstly found to play a central role in zebrafish embryonic development by (16, 17). Apela gene is located on chromosome and encodes a 54-amino acid preprotein, which is converted into a mature secretory form of 32-amino acid peptide after removal of the 22-residue signal peptide (17). The presence of cysteine residue in Apela following loss of signal peptide is an important feature that distinguish Apela and apelin (18) {Shin, 2017 #88; Pauli, 2014 #72; Shin, 2017 #88}. In zebrafish, it has been revealed that during development the Apela is expressed during gastrulation at the same time as APJ, whereas apelin is not expressed until the end of gastrulation (17). The spatiotemporal expression pattern of Aplea and APJ and receptor internalization experiments both indicate the possibility of Apela functioning as a ligand of APJ. Moreover, loss of Apela gene leaded to a phenotype similar to APJ gene deficiency, but different from APLN gene (19, 20). It has been demonstrated that Apela could activate APJ *in vitro* and apelin binding to APJ could rescue Apela deficiency (16, 17). It is considered that Apela/APJ signaling might be responsible for various physiological and pathological processes including blood pressure regulation, cardiac contractility, angiogenesis, fluid homeostasis, anti-renal fibrosis. Results suggest that Apela/APJ axis could guide angioblast to migrate to the midline to control vascular patterning in zebrafish embryos (21). Besides, losses of Apela could lead to preeclampsia and cardiovascular malformations (22).

Growing evidence has revealed the potential role of apelin/APJ system and Apela in various tumors in recent years (23, 24). It has been demonstrated that apelin expression is significantly increased in human non-small cell lung cancer (NSCLC) (25). The apelin expression correlated with poor overall survival (OS) and apelin could stimulate tumor growth and microvessel densities *in vivo* in NSCLC. Apelin gene upregulation was detected in half of the skin tumor samples (26). In postmenopausal breast cancer (BC) patients, high levels of apelin was observed (27). In several ovarian cancer subtypes, expression of Apela was elevated (28). These results indicate that apelin/APJ axis may play a critical role in the initiation and development of oncologic diseases. Hence, apelin/APJ axis and Apela can be considered as a therapeutic target in treating cancers. In this review, we would summarize the newest progression on the role of apelin/APJ system as well as Apela signaling in different cancers.

## Role of APELIN/APJ on Various Cancers

### Gastric and Gastroesophageal Cancer

For patients with advanced gastric cancer (GC), concurrent chemoradiotherapy (CRT) is the standard treatment (29, 30). However, response of this treatment is still low. Endostar is a modified recombinant human endostatin which could inhibit vascular endothelial growth factor (VEGF) expression in human GC cell line (31). However, effective marker of treatment response prediction is still lacking for GC patients receiving combination therapy of CRT and endostar. Hao et al. found that patients with high APJ expression had significant poorer response rate to combined therapy than patients with low APJ expression in the CRT plus endostar group (32). Patients with high APJ expression had significant low overall OS compared with those with low APJ expression. Hence, APJ can be used to predict treatment response in GC patients receiving combined therapy of CRT and endostar. In a study by Feng et al., it was revealed that tumor apelin levels, rather than serum apelin levels, correlated with advanced tumor stage, poor tumor differentiation, lymph node metastases and distant metastases. Compared with patients with low apelin levels and those with weak or negative apelin staining, patients with high tumor apelin levels showed a significantly shorter OS (33). The two studies indicated the important role of apelin/APJ axis in GC. Recently, Guan et al. discovered that circ-NOTCH1 could upregulate the expression of APLN by inhibiting the transcriptional activity of miR-637, leading to the regulation of cell growth in GC cell lines (34). This finding provided more evidence about the role of apelin in GC.

Contrary to the findings by Feng et al., in patients with gastroesophageal cancer (GEC), tumor apelin level as well as serum apelin level were increased compared with normal controls (35). Serum apelin levels positively correlated with high sensitive C-reactive protein (hsCRP) levels, indicating the potential role of apelin in systemic inflammatory response in GEC. These results suggested that apelin/APJ signaling may play a role in the progression of GC and GEC.

### Lung Cancer

It has been shown that compared with normal lung tissue, there was a significant increase in apelin mRNA levels in human NSCLC samples (25). High apelin protein levels correlated with elevated microvessel densities and poor OS, revealing the role of apelin in angiogenesis and clinical outcome of NSCLC. Yang et al. found that apelin-13 could promote lung adenocarcinoma cell proliferation and induced cell autophagy *via* extracellular signal–regulated kinases (ERK) 1/2 signaling (36). Ran et al. discovered that apelin could promote lung cancer A549 cells proliferation and invasion by inhibiting exosomal miR-15a-5p (37). Interestingly, a recent study revealed that miR-195 could directly target apelin, thus inhibiting lung adenocarcinoma cell proliferation and invasion (38). Lv et al. revealed that apelin-13 promoted lung adenocarcinoma cells migration by phosphorylating p21-activated kinase (PAK) 1 and cofilin, suggesting that apelin/APJ and downstream signaling may be potential therapeutic targets for anti-metastasis in patients with lung adenocarcinoma (39).

Recently, it was found that levels of serum apelin-12 were significantly higher in non-patient smokers than healthy non-smokers. Considering the subtype of lung cancers, the levels of serum apelin-12 were highest in patients with squamous cell carcinoma (SCC) compared to those with adenocarcinoma, small cell carcinoma and other malignancies (40). This result indicated that elevated levels of serum apelin-12 may contribute to the outbreak of lung SCC in non-patient smokers.

It has been proved that apelin plays an important role in angiogenesis in lung cancer. Uribesalgo et al. found that inhibition of apelin could lead to the modulating of tumor microenvironment, inhibition of tumor angiogenesis and tumor growth decreasing (41). By blocking apelin, it could prevent resistance and metastasis that induced by anti-angiogenic therapy. Prediction of clinical outcomes of patients with NSCLC treated with epidermal growth factor receptor-tyrosine kinase inhibitors (EGFR-TKIs) still remains difficult. Efforts are under way to develop potential biomarkers. Yang et al. found that compared with expression levels of apelin in the EGFR-TKIs sensitive group, there was a significant increase in the apelin expression in the EGFR-TKIs resistant group (42). High apelin expression may contribute to EGFR-TKIs resistance through modulating angiogenesis. The above studies indicated that apelin/APJ may contribute to lung cancer development and progression. Expression and role of apelin/APJ signaling in GC, GEC and lung cancer are summarized in **Table 1**.

**Table 1:** Expression and role of apelin/APJ signaling in gastric cancer, gastroesophageal cancer and lung cancer.

Disease	Tissue/cell line/serum	Protein	mRNA	Role	References
GC	Tissue	APJ ↑	–	–	Hao et al. (32)
	Tissue	Apelin ↑	–	Regulation of migration and invasion of GC cell lines	Feng et al. (33)
GEC	Tissue and serum	Apelin ↑	–	–	Diakowska et al. (35)
NSCLC	Tissue and cell line	Apelin ↑	Apelin ↑	Angiogenesis	Berta et al. (25)
Adenocarcinoma	Tissue Serum	APJ ↑ Apelin ↑	–	Promoting cell proliferation and autophagy via ERK1/2 signaling	Yang et al. (36)
Adenocarcinoma	Cell line	–	–	Promoting proliferation and invasion of cancer cells by inhibiting miR-15a-5p	Ran et al. (37)
Adenocarcinoma	Cell line	–	–	Promoting cells migration by phosphorylating PAK1 and cofilin	Lv et al. (39)
SCC	Cell line	Apelin ↑	–		Gholamnejad et al. (40)

GC, gastric cancer; GEC, gastroesophageal cancer; NSCLC, non-small cell lung cancer; SCC, squamous cell carcinoma; ↑, increase; -, not clear.

### Colon Cancer

Picault et al. revealed that apelin was overexpressed in human colon adenomas and adenocarcinomas (43). Overexpression of APJ was also observed in surrounding tissues. The proliferation rate of colorectal cancer cell lines was significantly reduced by treating them with apelin-13 receptor antagonist. These findings showed us the potential of apelin/APJ signaling as therapeutic target for colon cancer treatment. Similarly, Liu et al. discovered that elevated apelin expression was found in colon tumor compared with adjacent non-tumor tissue in the same patient (44).

Chen et al. found that apelin-13 could stimulate Notch3 expression in colorectal cancer cell line LS180. LS180 proliferation was inhibited by blocking either APJ or Notch3, suggesting that apelin-13/APJ could promote colorectal cancer cell proliferation by modulating Notch3 pathways (45). In a study by Podgórska et al., four apelin peptides, including [Pyr1] apelin-13, apelin-13, apelin-17, and apelin-36, could promote colon cancer cells migration and invasion (46).

As an anti-angiogenesis monoclonal antibody, bevacizumab has been used in several cancers. However, only 10% to 15% of patients with colorectal cancer could benefit from bevacizumab therapy (47). Thus, biomarkers that can predict treatment response of bevacizumab in colorectal cancer patients are of urgent need. Zuurbier et al. found that expression of apelin mRNA was significantly higher in bevacizumab non-response group compared to bevacizumab response group (47). Besides, overexpression of apelin protein was associated with poor progression-free survival in patients receiving bevacizumab treatment. Thus, apelin may be used as a novel biomarker in predicting bevacizumab response in patients with colorectal cancer. These results showed us the ability of apelin/APJ signaling in colon cancer development. Expression and role of apelin/APJ signaling in colon cancer are summarized in **Table 2**.

**Table 2:** Expression and role of apelin/APJ signaling in colon cancer.

Disease	Tissue/cell line/serum	Protein	mRNA	Role	References
Adenocarcinoma	Tissue and cell lines	Apelin ↑ APJ ↑	–	Protecting colon cancer cells from apoptosis by inactivating a caspase-dependent pathway and decreasing the degradation of PARP	Picault et al. (43)
Adenocarcinoma	Tissue	Apelin ↑	–		Liu et al. (44)
Adenocarcinoma	Tissue and cell lines	Apelin ↑ APJ ↑	–	Promoting proliferation of colon carcinoma cells by activating Notch3 signaling pathway	Chen et al. (45)
Adenocarcinoma	Cell line	–	–	Effecting Migration and Invasion of Colon Cancer Cells through several possible mechanisms	Podgórska et al. (46)
Adenocarcinoma	Tissue	Apelin ↑	Apelin ↑		Zuurbier et al. (47)

↑, increase; -, not clear.

### Brain Tumor

Glioma is most common malignant tumor in the brain with a poor prognosis. Treatment for glioma still remains difficult. In a study by Kälin et al., expression of apelin and APJ was elevated in microvascular proliferations of malignant gliomas (48). Zhang et al. revealed that circular RNA circ-ZNF264 can upregulate miR-4493 target gene apelin expression, leading to glioma cell proliferation, invasion and inhibition of apoptosis (49).

Glioblastoma is a highly aggressive brain tumor with an extremely poor prognosis. Glioblastoma stem-like cells (GSCs), a subpopulation of tumor-initiating cells, have played a critical role in tumor initiation, tumor invasion, angiogenesis and treatment resistance (50–52). GSCs reside closely to vascular beds, into which endothelial cells secrete unknown factors that can regulate their plasticity, survival and fate (53, 54). Wright et al. found that endothelial cells could produce apelin (53, 54). *In vitro*, apelin could increase GSC expansion (53). Both *in vitro* and *in vivo*, inhibition of APJ could lead to a significant reduction in tumor growth (53). The study by Frisch et al. showed a similar result (55). Knockdown of APLN resulted in reduction of tumor vasculature in glioblastoma (55). The results of these studies undercover the potential of apelin/APJ signaling as a drug target for glioblastoma. Mastrella et al. discovered that compared to controls, knockdown or knockout of APLN in orthotopic models of proneural or classical glioblastoma subtypes contributed to a significant reduction in glioblastoma vascularization (56). However, apelin expression reduction was found to promote glioblastoma cell invasion. These results indicated the dichotomous roles of apelin/APJ signaling in tumor invasion and angiogenesis in glioblastoma. Besides, the authors also discovered that apelin-F13A could bind to APJ, leading to the inhibition of glioblastoma cell invasion and tumor angiogenesis. All these results revealed that apelin/APJ axis may play an important role in brain tumor.

### Hepatocellular Carcinoma

Cancer-associated fibroblast (CAF) plays an important role in promoting cancer cells invasion. CAF could cause up-regulation of some genes in hepatocellular carcinoma (HCC) cells (57). However, it still requires investigation on how HCC cells could affect CAF gene expression. It was found that human HCC cell line HCC38/KMUH could upregulate APLN in CAF cell line F26/KMUH. Muto et al. demonstrated that apelin/APJ axis was overexpressed in HCC and could regulate angiogenesis in HCC (58). In another study, apelin was found to have the potential to transform epithelial cells and initiate HCC progression in hepatitis-C virus (HCV) chronic hepatitis (59).

Cabiati et al. revealed that expression of apelin/APJ axis was significantly elevated in liver recipients compared with liver donors from liver specimens obtained from HCV-positive HCC who underwent liver transplantation (60). Lee et al. discovered that high APJ expression correlated with presence of microvascular invasion, intrahepatic metastasis and early recurrence in HCC (61). Multivariate analysis revealed that high APJ expression independently predicted a shorter recurrence-free survival in HCC. In a recent study focusing on role of apelin in HCC, the findings showed that by binding to APJ, apelin could activate phosphatidylinositol 3-kinase (PI3K)/Akt pathway, resulting in an increase in expression of phospho-glycogen synthase kinase 3β (p-GSK3β) and cyclin D1 (62). ML221, an APJ antagonist, could inhibit Apelin-PI3K/Akt signaling pathway and HCC growth both *in vitro* and *in vivo.* In a study by Huang et al., it demonstrated that apelin-13 may induce autophagy in HCC cell line HepG2 cells by activating ERK1/2 and upregulating Beclin1 expression (63). Apelin/APJ may play critical roles in HCC development. Expression and role of apelin/APJ signaling in brain tumor and HCC are summarized in **Table 3**.

**Table 3:** Expression and role of apelin/APJ signaling in brain tumor and HCC.

Disease	Tissue/cell line/serum	Protein	mRNA	Role	References
Malignant glioma	Tissue	Apelin ↑ APJ ↑	–		Kälin et al. (48)
Glioblastoma	Tissue	–	Apelin ↑		Harford-Wright et al. (53)
Glioblastoma	Tissue	Apelin ↑ APJ ↑	–	Tumor angiogenesis	Frisch et al. (55)
Glioblastoma	Tissue	Apelin ↑ APJ ↑	–	Dichotomous roles of apelin/APJ signaling in tumor invasion and angiogenesis in glioblastoma.	Mastrella et al. (56)
HCC	Tissue and cell line Tissue	- APJ ↑	Apelin ↑ -		Muto et al. (58)
HCC	Tissue	APJ ↑	–		Lee et al. (61)
HCC	Tissue Cell line	Apelin ↑ Apelin ↑	Apelin ↑ -	Apelin could activate PI3K/Akt pathway, resulting in an increased expression of p-GSK3β and cyclin D1	Chen et al. (62)
HCC	Cell line	–	–	Apelin may promote autophagy in HCC cells by activating ERK1/2 and upregulating Beclin1expression.	Huang et al. (63)

HCC, hepatocellular carcinoma; ↑, increase; -, not clear.

### Genitourinary Cancers and Pelvic Cancers

Apelin/APj axis was also found to play an important role in genitourinary cancers. Zhang et al. found that apelin mRNA expression was up-regulated in clear cell renal cell carcinoma (ccRCC) specimens from the GSE6344 microarray dataset (64). However, there was no significant difference between ccRCC tissue and adjacent normal tissues in apelin mRNA expression obtained from their center (64). Levels of plasma apelin were elevated in patients with hyponatremia, which correlated with a greater risk of cancer progression and death (65). Tolkach et al. found that APJ mRNA expression was significantly associated with tumor aggressiveness in ccRCC. APJ expression was negatively associated with programmed cell death-ligand 1 (PD-L1) expression in ccRCC cells in a subset ccRCC of patients (66). In muscle-invasive bladder cancer, apelin protein expression level was elevated in tumor tissues compared with adjacent normal tissues (67). High expression of apelin significantly correlated with high tumor stage, distant metastasis, vascular invasion and may have the potential to indicate poor prognosis. In prostate cancer (PCa), Wan et al. found that microRNA miR-224 could target apelin gene. In PCa tissues, down-regulation of miR-224 was negatively associated with the up-regulation of apelin mRNA (68). Furthermore, upregulation of apelin was more discovered in PCa with advanced stage, metastasis and prostate-specific antigen failure. It was also revealed that apelin-13 could promote human PCa cells LNCaP proliferation through an androgen receptor-dependent manner (69).

In a study by Altinkaya et al., serum levels of apelin were higher in patients with endometrial cancer than controls (70). Apelin levels were significantly associated with body mass index (BMI). High apelin levels were found to be associated with an increased risk of endometrial cancer. Dupont et al. discovered that adipokines, including apelin, may activate different signaling pathways like PI3K/Akt, AMP-activated protein kinase (AMPK), and peroxisome proliferator-activated receptor (PPAR) that might contribute to the development of ovarian cancer (71). In ovarian cancer, APJ expression levels were higher in epithelial cancer cells than in granulosa tumor cells and apelin could act as a mitogenic factor in ovarian cancer cell line OVCAR-3 cell, promoting its proliferation and growth (72). In another study, it was revealed that compared to controls, APJ overexpressed tumors showed reduced response to sorafenib treatment in xenograft model in ovarian cancer (73). In genitourinary cancers and pelvic cancers, the apelin/APJ signaling may have important impact.

### Other Types of Cancers

In patients with multiple myeloma, levels of plasma apelin were significantly elevated compared with those in non-Hodgkin lymphoma and healthy controls (74). Patients with high apelin level had a better prognosis. Heo et al. demonstrated that expression of apelin was significantly associated with tumor recurrence and disease-free survival in oral squamous cell carcinoma (OSCC) (75). Under hypoxic conditions, apelin expression was upregulated and exogenous apelin could stimulated the proliferation and migration of oral cancer cell line HSC-3 through phosphorylation of ERK1/2. The results suggest the potential of hypoxia-induced apelin as a new therapeutic target for OSCC. Apelin/APJ expression was also significantly increased in pancreatic adenocarcinoma (26, 73). In cholangiocarcinoma, expression of the apelin/APJ was increased. *In vitro*, apelin could promote human cholangiocarcinoma cells Mz-ChA-1 proliferation and angiogenesis which could be inhibited by ML221. Growth of xenograft mice was inhibited by ML221 treatment (76). In postmenopausal patients with breast cancer, an increase in level of serum apelin-36 was detected and was positively associated with BMI (27). These results indicated that apelin and APJ may play important roles in different types of cancers. Expression and role of apelin/APJ signaling in genitourinary cancers, pelvic cancers and other types of cancer are summarized in **Table 4**.

**Table 4:** Expression and role of apelin/APJ signaling in genitourinary cancers, pelvic cancers and other types of cancers.

Disease	Tissue/cell line/serum	Protein	mRNA	Role	References
ccRCC	Tissue	–	Apelin *		Zhang et al. (64)
Muscle-invasive bladder cancer	Tissue	Apelin ↑	–		Yang et al. (67)
PCa	Tissue	–	Apelin ↑		Wan et al. (68)
Obesity-related Endometrial cancer	Serum	Apelin ↑	–		Altinkaya et al. (70)
Ovarian cancer	Cell line	Apelin ↑ APJ ↑	–	Apelin could act as a mitogenic factor in ovarian cancer cell line, promoting its proliferation and growth	Hoffmann et al. (72)
Multiple myeloma	Serum	–	Apelin ↑		Maden et al. (74)
OSCC	Tissue Cell line	Apelin ↑ Apelin ↑	- Apelin ↑	Apelin could stimulated the proliferation and migration of oral cancer cell line through phosphorylation of ERK1/2	Heo et al. (75)
Pancreatic adenocarcinoma	Tissue	Apelin ↑ APJ ↑	Apelin ↑		Sorli et al. (26)
Cholangiocarcinoma	Tissue Cell line	APJ ↑ Apelin ↑ APJ ↑	Apelin ↑ APJ ↑		Hall et al. (76)

PCa, prostate cancer; OSCC, ovarian clear cell carcinoma; ↑, increase; -, not clear; *, positive result.

### Role of Apela in Cancers

Despite the various roles of Apela in biological functions, role of Apela in cancers is poorly understood. Recent years saw a significant increase in the studies focusing on the association between Apela and cancer. Soulet et al. found that Apela was usually expressed by epithelial cells while expression of APJ could be found in various kidney cells (77). Expression of Aplea was reduced in the main kidney cancer subtypes, namely, clear cell, papillary, and chromophobe RCC. In renal cancer cells, Apela could induced apoptosis and inhibited cell proliferation and migration through activating mTORC1 signaling. These results suggest the potential role of Apela in renal cell treatment. In another study by Artas et al., similar expression pattern of Apela was discovered. In chromophobe RCC, expression of Apela was significantly reduced compared to health controls while no expression of Apela was detected in papillary RCC or Fuhrman grade 1 and grade 2 ccRCC (78). However, there existed a significant increase in the expression of Apela in renal oncocytoma compared with health controls. Hence, Apela may be useful in differentiating benign and malign renal tumors.

In brain tumor patients, it was found that Apela mRNA and protein were both expressed at high levels in a subset of patients (79). High Apela expression correlated with poor survival in patients with glioma and glioblastoma and Apela expression was associated with glioma grade. However, gene expression of apelin or APJ was not found to be associated with patient survival or glioma grade. Artas et al. also discovered that there existed a significant difference in Apela expression between control tissues and glioma tissues (80). In high-grade glioma, expression of Apela was significantly higher than that in low-grade glioma.

In TMPRSS2-ERG subtype of locally advanced lymph node-positive PCa, Apela gene was identified to be associated with prognosis (81). In ovarian cancer, expression of Apela was elevated in various ovarian cancer subtypes, especially in ovarian clear cell carcinoma (OCCC) (28). APELA could promote OCCC cell line OVISE proliferation and migration *in vitro*. Furthermore, it was revealed that regulation of OCCC cell growth by Apela depended on p53 activity. These results uncovered different roles of Apela in different types of cancers including RCC, brain tumor and ovarian cancer. Expression and role of Apela in different types of cancers are summarized in **Table 5**.

**Table 5:** Expression and role of Apela signaling in different types of cancers.

Disease	Tissue/cell line/serum	Protein	mRNA	Role	References
RCC	Tissue	Apela↓	Apela↓	Inducing apoptosis and inhibiting proliferation and migration through activating mTORC1 signaling in renal cancer cells	Soulet et al. (77)
Brain tumor	Tissue	Apela ↑	Apela ↑		Ganguly et al. (79)
OCCC	Tissue Cell line		Apela ↑ Apela ↑	Promoting OCCC cell line proliferation and migration	Yi et al. (28)

RCC, renal cell carcinoma; OCCC, ovarian clear cell carcinoma; ↑, increase; ↓, reduce.

## Discussion

**Figure 1:**
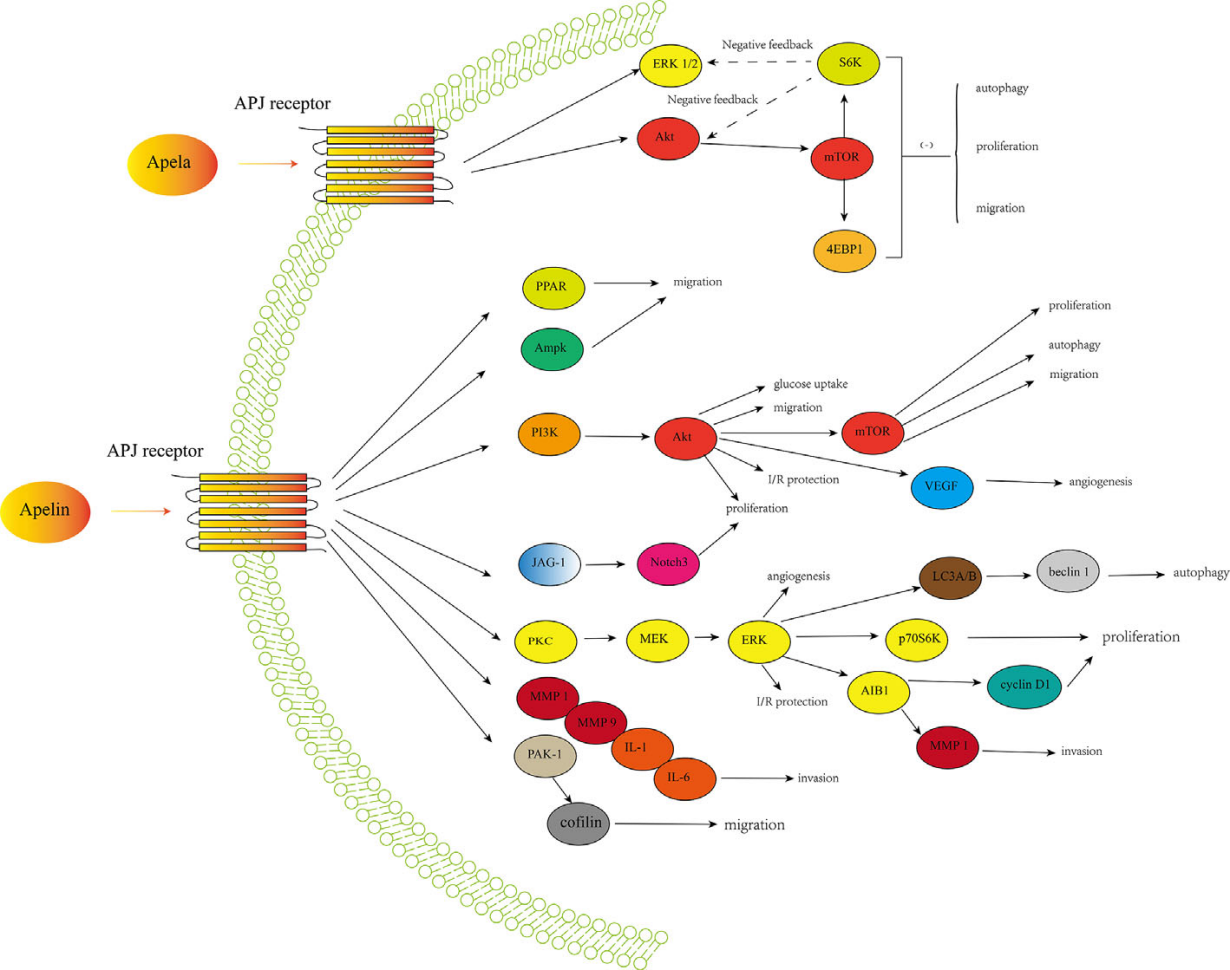
A simple overview of the apelin/APJ-induced signaling pathways and Apela/APJ-induced signaling pathways involved in cancer development and progression.

Expression of apelin/APJ signaling occurred in many cancers, suggesting a potential role of this axis in cancer development and progression. Apelin is involved in cancer cell proliferation in various cancers, including NSCLC, GC, oral squamous cell carcinoma, cholangiocarcinoma, PCa and ovarian cancer. It was revealed that apelin could promote cancer cell proliferation by increasing expression of factors involved in cell proliferation including cyclin D1, Ki-67, proliferating cell nuclear antigen (PCNA), and activating pathways like JAG1/Notch3, ERK1/2, and PI3K/Akt (73). Apelin could also increase cell migration in lung adenocarcinoma, GC and oral squamous cell carcinoma. There are several possible signaling pathways and factors involved in cell migration including PAK1/cofilin, mitogen-activated protein kinase (MAPK)/ERK, AMPK, PI3K/Akt, PPAR, bone morphogenetic protein (BMP)-2, focal adhesion kinase (FAK), matrix metalloproteinase (MMP), osteopontin (OPN), platelet derived growth factor (PDGF), a disintegrin and metalloproteinase with thrombospondin motifs (ADAMTS) and stromal cell-derived factor-1 alpha (SDF-1α)/CXC chemokine receptor-4 (CXCR4) (69, 73). The role of apelin/APJ signaling in contributing to angiogenesis is also well-recognized in various cancers (69, 73). Besides, the anti-apoptosis function of apelin is also well-described (82, 83). In several cancers, apelin could also protect cancer cells from apoptosis and increases cancer stability (43, 53). It has been revealed that apelin/APJ may also play a role in mediating differentiation of mesenchymal stem cells to cancer stem cells (73). Besides, apelin/APJ signaling may play a critical role in cancer stem cells self-renew by activating signaling pathways like wnt/β-catenin and Jagged/Notch (73). Furthermore,several studies have shown the pivotal role of apelin/APJ signaling in leading to resistance to anticancer drugs, especially chemotherapy and anti-angiogenic drugs (32, 39, 73). **Figure 1** showed a simple overview of the apelin/APJ and Apela/APJ axis induced signaling pathways involved in cancer.

Compared to apelin, the role of Apela in cancer is not well studied. Researches are still under investigation. In renal cancer, Apela could induced apoptosis and inhibited cell proliferation and migration while Apela could promote ovarian clear cell carcinoma cell line proliferation and migration. The reason for the opposing roles of Apela in different cancers still remains unclear. Ovarian cancer cell line OVISE does not express APJ, suggesting that Apela may function in an APJ-independent pathway (28). Further investigations are needed.

The apelin, APJ and Apela are detected in many types of cancer. In most situations, expression levels of apelin, APJ and Aplea are elevated compared to health controls. Moreover, the apelin/APJ and Apela/APJ signaling contributed to cancer development and progression, indicating the potential of signaling as therapeutic targets for cancer treatment. However, the underlying mechanisms are still unclear. More studies should be carried out to get a better understanding of the function of apelin/APJ and Apela signaling in cancer.

## Author Contributions

XG conceived and designed the idea, reviewed the manuscript. LL and XY wrote the manuscript. CL and YW completed the figure. QX, LZ, and YP prepared the tables. All authors contributed to the article and approved the submitted version.

## Funding

This study was supported by the National Natural Science Foundation of China (81902727).

## Conflict of Interest

The authors declare that the research was conducted in the absence of any commercial or financial relationships that could be construed as a potential conflict of interest.
